# *The Organon* and Materia Medica Club of the Bay Cities of California

**Published:** 1896-03

**Authors:** W. E. Ledyard

**Affiliations:** Secretary


W. E. LEDYARD, M. D., *Secretary*.

Reported by Eleanor F. Martin, M. D.

---

### *THE ORGANON* AND MATERIA MEDICA CLUB OF THE BAY CITIES OF CALIFORNIA.

The regular semi-monthly meeting was held at the office of Dr. M. T. Wilson, 824 Ellis Street, San Francisco, Friday evening, September 20th, 1895.

Members present: Drs. J. M. Selfridge, M. T. Wilson, A. McNeil, W. E. Ledyard, E. W. Bradley, and George H. Martin.

The meeting was called to order at 8.15 by the President, Dr. J. M. Selfridge. The minutes of the previous meeting were read by the Secretary, Dr. W. E. Ledyard, and after slight corrections by Drs. McNeil and Selfridge, were approved.

Dr. McNeil presented a favorable report from the Board of Censors regarding the name of Dr. M. F. Underwood for membership.

Dr. Martin moved that the report be adopted and that Dr. Underwood be elected a member. Dr. Wilson seconded this motion, adding that the candidate be balloted for. Dr. Martin moved that the Secretary cast the ballot for the candidate, which was seconded by Dr. Wilson. The Secretary then cast the ballot for Dr. Underwood, electing him a member of the Club, the Secretary being instructed to notify him of the fact.

The President then made a few suggestions regarding the discussions and minutes of the various meetings, stating that he thought the members should endeavor to be as brief as possible in their remarks, simply bringing out the points they wished to discuss, without any superfluous remarks. He also suggested that the reporter leave out any superfluous matter spoken of at the meetings and report only such matter as related exclusively to the points under discussion. It was agreed by all that the reporter use her own judgment in reporting what she considered of the most importance.

As the reading of *The Organon* was soon to be completed, the question of the advisability of commencing *The Organon* again or taking up some other work was discussed. The President suggested that as some of the members did not have copies of the *Chronic Diseases*, the book they wished to take up next, *The Organon* be again taken up until the new publication of the *Chronic Diseases* was out. Dr. Wilson made a motion to this effect, which was seconded by Dr. Martin, and carried.

Dr. McNeil was then appointed reader of the evening, and commenced *The Organon* at Section 252.

### Discussion.

Dr. McNeil—There are chronic diseases that are not psoric.

Dr. Selfridge—Hahnemann says that psora is the basis of all chronic diseases.

Dr. McNeil—One of the most important remedies in chronic rheumatism is *Rhus*, and it is not anti-psoric. *Pulsatilla* is often indicated in dysmenorrhœa, and most frequently cures, and yet it is not an anti-psoric remedy. There are many diseases chronic and yet are not necessarily psoric; as, for instance, chlorosis.

Dr. Ledyard—In a chronic case, acute symptoms may come up and you may have to use another remedy for them. In a case of cancer under my care *Belladonna* is at present the indicated remedy, which I am giving. I do not think this remedy will cure the disease, but I give it to relieve the acute symptoms that are prominent at present.

Dr. McNeil—An anti-psoric remedy is necessary to cure in such a case. *Belladonna* and *Calcarea-carb.* are complementary remedies. *Belladonna* will cure certain symptoms arising in such a case, but will not cure the case. *Calcarea-carb.* will come in good later. *Pulsatilla* and *Sulphuric acid* are also complementary remedies. These remedies come in almost alternately in the Hahnemannian sense.

Section 253.

DISCUSSION.

Dr. Selfridge—This seems to be a distinction without a difference.

Dr. McNeil—Instead of giving a teaspoonful we should give a few small pellets. This is what he means. He used the pellets because he thought it the smallest dose. If given in solution he thought it would be too large a dose.

Sections 254 and 255.

DISCUSSION.

Dr. McNeil—This, I confess from experience, is hard to do; to see whether the drug is doing the work or not and how long to wait. The time varies according to the disease and to the remedy employed. It is evidenced by the improved mental condition before a change in the physical condition is noticed.

Sections 256 and 257.

DISCUSSION.

Dr. McNeil—Professor Hempel has given seventeen lectures upon *Aconite*, and when they are all summed up we find that he tells us to give *Aconite* for all diseases, and if it does not cure to give it stronger.

Dr. Selfridge—This is a caution not to fall into ruts in the practice of medicine, as we are apt to do.

Sections 258–260.

DISCUSSION.

Dr. Selfridge—Hahnemann has omitted one thing here which undoubtedly interferes with the action of remedies—tobacco. It is extremely deleterious to the human heart, especially weak hearts and weak stomachs. In a case of mine of a man with bad digestion who was in the habit of using tea, coffee, and tobacco, he had *great distress one hour after eating.* I gave him one prescription of *Nux-vom.*$^{200}$, and told him to stop the use of the tea, coffee, and tobacco. He was cured, but I am sure would not have been had he kept on with the tea, coffee, and tobacco.

Dr. Martin—I have found this to be a fact. Men who were all whisky or tobacco-soaked. They were dependent upon these stimulants, and they could not be taken from them. Yet when the indicated remedy was given it would act satisfactorily.

Dr. Selfridge—Doubtless this was so when the disease was not produced by the whisky or tobacco. If the disease is caused by these they should be stopped.

Dr. Martin—No; the diseases in the cases I refer to were not caused by these stimulants.

Sections 261 and 262.

### Discussion.

Dr. McNeil—The question of hygiene is precisely in the same position to-day that the question of therapeutics was one hundred years ago. We have eminent authorities on the other side for every point made by hygienists. There is a confused state regarding hygiene at the present time. Why should this be so? Because there is need for a law to govern hygienic conditions—as in therapeutics before Hahnemann's time. The eminent men of the time saw that a change was needed; that there was a necessity for a law to govern therapeutics. Even Hippocrates saw it; and Hahnemann put their thoughts into action. As to diet, has it not often been proved that that which we crave is wholesome, and that which is disagreeable to us is unwholesome. A sick dog or cat will eat grass because it is what they require. If we require *Natrum-mur.* we have a craving for salt. The law here is that the satisfying of our craving is beneficial. Hahnemann limits this, however, to acute diseases.

Dr. Bradley—There are times in diseases when patients crave things that are harmful.

Dr. Selfridge—The physician must decide what is harmful; as for instance, when a patient craves chalk, charcoal, slate-pencils, etc.

Dr. McNeil—There is a want in the system for certain things. The thing to do in such cases is to give them all they want, and also the indicated remedy, and it will stop the craving. There is no limit as regards acute or chronic diseases. What prevails in acute diseases may also prevail in chronic diseases. As to vices; tobacco-users, etc. Nature has long been accustomed to certain stimulants, even for fifty years. On the one hand, gratification is habit; and on the other hand, there is the hurtful influence of this habit. The question is, which will do the most harm? To let the patient go on, or to break him of this habit? It might be almost certain death to stop it.

Dr. Selfridge—It is best to diminish the quantity of the stimulant. In a case of amaurosis caused by tobacco, it was cured by simply stopping the use of the drug, showing that nature throws off the effects of the drug.

Dr. Bradley—In cholera morbus there is frequently great thirst for cold water. Sometimes a teaspoonful will immediately cause vomiting and purging, and you cannot allow the patient to have the water no matter how much he may crave it.

Dr. McNeil—In an Arsenical condition for instance. Should we give the small quantities of water craved for or deprive the patient of it? In my early practice I did limit my patients. Now I give them all they want. In its course through the alimentary canal some of it may be absorbed.

Dr. Bradley—What would you do in a case of dysentery if it made the patient worse?

Dr. McNeil—I would give it.

Section 263.

### Discussion.

Dr. McNeil—On this point of temperature. I had a case in Michigan of an old lady seventy years of age with a clear case of cholera morbus. She was very sick, but insisted on sitting with an open door and window on each side of her. Outside there was a cold, raw, west wind blowing. We granted her wish; gave her *Carbo-veg.*, and carried her through all right. I doubt if *Carbo-veg.* would have cured her without the large amount of oxygen which she craved.

Dr. Ledyard—I think in Dr. McNeil's case the fresh air supplied to the vital forces what was needed.

Dr. Bradley—I think a good deal of this craving is habit. I cannot see that this woman received any more oxygen than she would if she had not been in a draught.

Dr. McNeil—Suppose that woman had been kept in a room of seventy degrees pure air. Every breath would be taking in oxygen, but it would be rarified by the temperature.

Dr. Bradley—I do not believe the blood took up any more oxygen even though the lungs took it in.

Dr. Selfridge—I think Dr. McNeil did right. She needed the oxygen as one needs a pickle who craves it while suffering from dysentery.

This ended the discussion of *The Organon*, and Dr. Selfridge then said: I wish to relate a beautiful effect of a remedy in a patient of mine—a young lady who suffered intensely with every menstruation. Her mother told me it was paroxysmal. About two months ago I found out the key-note. She menstruated usually about one week over her time. At the time she would be troubled with a cold, icy perspiration, especially on the face and forehead, with considerable prostration. I prescribed Veratrum-alb.$^{200}$, and the pain became immensely less. The next time she had not a particle of pain, and the flow came a little early. Another case. Young lady, twenty years of age, who had been under the care of a gynæcologist at Fabiola Hospital when she was eighteen years old. She had been curetted, and a capital operation was about to be performed to remove the right ovary, when the mother interfered, and it was not done. She had been an invalid for these two years, and was at last brought to me. Key-note—color dark; no pain until the flow commenced, when it was very intense, and lasted until the flow was well established, when it ceased. I prescribed *Thuja*. Every symptom is better, though not entirely gone. She will report after the next period, and I expect her to be cured.

Upon motion of Dr. Wilson, seconded by Dr. Martin, the meeting then adjourned to meet the first Friday in October at the office of Dr. A. McNeil, 784 Van Ness Avenue, San Francisco, when the reading of *The Organon* would be commenced at Section 264.

